# MicroRNAs as potential architects of immune dysregulation and megakaryocytic failure in immune thrombocytopenia

**DOI:** 10.1111/bjh.70485

**Published:** 2026-04-15

**Authors:** Zhenyu Liu, Johan Rebetz, Lamya Garabet, Waleed Ghanima, Peipei Xu, Hans Wadenvik, Drew Provan, Rick Kapur, John W. Semple

**Affiliations:** ^1^ Division of Transfusion Medicine, Department of Laboratory Medicine Lund University Lund Sweden; ^2^ Center for Laboratory Medicine Østfold Hospital Trust Grålum Norway; ^3^ Multidisciplinary Laboratory Medicine and Medical Biochemistry Akershus University Hospital Lørenskog Norway; ^4^ Department of Research Østfold Hospital Trust Grålum Norway; ^5^ Department of Medicine Østfold Hospital Trust Grålum Norway; ^6^ Institute of Clinical Medicine University of Oslo Oslo Norway; ^7^ Department of Hematology, Nanjing Drum Tower Hospital, Affiliated Hospital of Medical School Nanjing University Nanjing China; ^8^ Department of Internal Medicine and Clinical Nutrition Sahlgrenska Academy Gothenburg Sweden; ^9^ Department of Haematology, Blizard Institute, Barts and The London School of Medicine and Dentistry Queen Mary University of London London UK; ^10^ Sanquin Blood Supply Foundation, Department Research, and Amsterdam UMC Location University of Amsterdam Landsteiner Laboratory Amsterdam The Netherlands; ^11^ Department of Clinical Immunology and Transfusion Medicine Office of Medical Services Lund Sweden; ^12^ Department of Pharmacology, Medicine and Laboratory Medicine and Pathobiology University of Toronto Toronto Canada

**Keywords:** immunity, immunoregulation, ITP, microRNA (miRNA)

## Abstract

Immune thrombocytopenia (ITP) is a complex autoimmune disorder characterized by accelerated destruction of peripheral platelets and impaired megakaryopoiesis. While the cellular effectors, dysregulated T cells, hyperactive B cells and phagocytic macrophages are well characterized, the upstream epigenetic mechanisms orchestrating this multicellular immune network remain largely elusive. This review explores the hypothesis that microRNAs (miRNAs) may serve as critical architects of immune dysregulation and bone marrow failure in ITP. We evaluate the clinical utility of circulating miRNAs as non‐invasive biomarkers for diagnosis, risk stratification and predicting response to steroid and thrombopoietin receptor agonists therapies. Finally, we address current translational difficulties, such as data fragmentation and pre‐analytical variables. We propose a roadmap for integrating functional validation with multi‐omics, utilizing miRNA‐based approaches to facilitate and advance precision medicine in ITP.

## THE IMMUNOPATHOGENIC LANDSCAPE OF ITP: A DYSREGULATED MULTICELLULAR NETWORK

Immune thrombocytopenia (ITP) is an acquired autoimmune bleeding disorder characterized by isolated thrombocytopenia, commonly defined as a platelet count <100 × 10^9^/L, and diagnosed after excluding alternative causes of thrombocytopenia.^1^, ^2^, ^3^ ITP can be primary or secondary. Secondary ITP is usually associated with infections, autoimmune diseases, lymphoproliferative disorders, drugs and other conditions.^4^, ^5^ Epidemiologic studies suggest that about two to four new cases occur among 100 000 adults every year.^3^ The clinical course varies, with nearly 70% of adult patients developing a persistent or chronic condition. The goals of ITP therapy are to prevent clinically significant bleeding, achieve and maintain the platelet count and improve quality of life while minimizing treatment toxicity. First‐line therapy in adults is typically corticosteroid‐based, with intravenous immunoglobulin (IVIG) used when a rapid platelet rise is needed or in specific high‐risk settings.^6^ For patients requiring subsequent therapy, standard second‐line options include thrombopoietin receptor agonists (TPO‐RAs), rituximab and splenectomy, with shared decision‐making between doctors and patients emphasized in contemporary guidance.^7^, ^8^, ^9^ ITP immunopathogenesis is commonly conceptualized as a dysregulation of B‐ and T‐cell homeostasis leading to a dual process involving increased peripheral platelet destruction together with impaired platelet production in the bone marrow.^6^, ^10^, ^11^, ^12^, ^13^, ^14^, ^15^

Following a breakdown of immune tolerance, antigen‐presenting cells can present platelet‐derived autoantigens to autoreactive T cells, leading to effector T‐cell activation and the provision of help to B cells, thereby initiating and sustaining autoimmunity.^16^ Systematic reviews and mechanistic studies describe a skewing of CD4^+^ T‐cell responses towards T helper cell 1/T helper cell 17 (Th1/Th17)‐like effector phenotypes with accompanying pro‐inflammatory cytokine changes,^17^ alongside quantitative and/or functional defects in regulatory T cells (Tregs).^18^, ^19^ The enhanced effector responses coupled with weakened immune regulation create a permissive environment for both humoral and cytotoxic immune injury towards platelets. Against this background, B cells become activated and differentiate into plasma cells, producing platelet‐autoantibodies directly against platelet surface glycoproteins. Autoantibody‐opsonized platelets are then cleared via Fcγ receptor (FcγR)–mediated phagocytosis by macrophages in the spleen and liver by antibody‐dependent cellular phagocytosis (ADCP).^20^ Platelet autoantibodies play an important role in the mechanism of ITP, yet the monoclonal antibody‐specific immobilization of platelet antigens (MAIPA) assay, which is currently considered the gold standard of autoantibody testing, has limited sensitivity.^21^, ^22^ It serves as a valuable rule‐in test, but the discovery of better disease‐specific biomarkers is required. ITP remains largely a diagnosis of exclusion in routine clinical practice. Beyond humoral immunity, cellular immunity can also contribute to thrombocytopenia. Recent studies support the concept of antigen‐driven clonal expansion of cytotoxic CD8^+^ T cells in subsets of patients, with evidence that these T cells can interact with autologous platelets and induce platelet activation and apoptosis.^23^ In addition, cytotoxic T cells appear to target bone marrow megakaryocytes, contributing to megakaryocyte injury and impairing platelet production. Therefore, ITP is not solely a disorder of platelet clearance but also involves defective platelet production.

**FIGURE 1:**
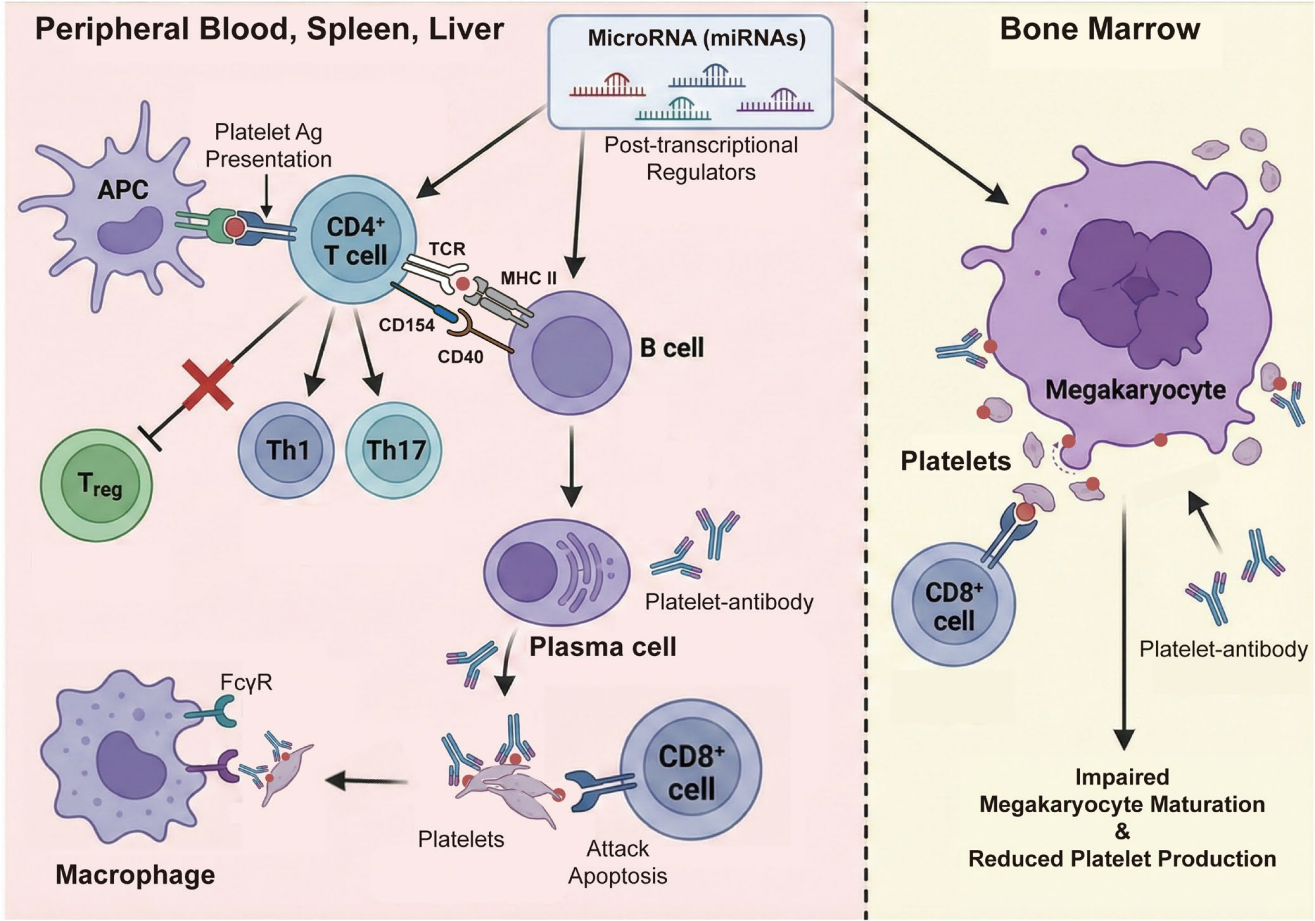
Schematic representation of the immunopathogenesis of immune thrombocytopenia (ITP). The figure depicts the breakdown of immune tolerance in peripheral blood and lymphoid organs (left) and the bone marrow niche (right). The pathology of ITP involves the expansion of Th1 and Th17 cells and the dysfunction of Tregs. This T‐cell imbalance drives B cells to differentiate into plasma cells and secrete platelet autoantibodies, which bind to platelet antigens and trigger FcγR‐mediated phagocytosis by macrophages. Additionally, aberrant CD8^+^ T cells can attack platelets directly. In the bone marrow microenvironment, platelet antibodies bind to megakaryocytes, resulting in megakaryocyte dysfunction and suppressed platelet production. Notably, dysregulated microRNAs (miRNAs) play a pivotal role in this process by modulating the functions and subtypes of various immune cells, including pro‐inflammatory Th1/Th17 subsets, defective Treg, increased B‐cell survival, the upregulation of FcγR‐dependent macrophage phagocytosis. Ab, antibody; Ag, antigen; APC, antigen‐presenting cell; CD, cluster of differentiation; FcγR, Fc gamma receptor; MHC, major histocompatibility complex; miRNA, microRNA; TCR, T‐cell receptor; Th, T helper cell; Treg, regulatory T cell.

Collectively, ITP can be viewed as a multicellular immune network disorder; dysregulated T‐cell regulatory balance facilitates the initiation and maintenance of autoimmunity; autoreactive plasma cells generate platelet antibodies that promote FcγR‐dependent macrophage clearance of opsonized platelets; and cytotoxic immune injury, together with bone marrow megakaryocyte dysfunction, contributes to impaired platelet production (Figure 1). However, the upstream regulatory layer that orchestrates these complicated immune circuits has still not been clearly identified. In addition, the potential of these molecular targets for patient stratification, response prediction and therapeutic intervention also remains to be determined. Indeed, accumulating evidence from high‐throughput profiling, predominantly microarray and small ribonucleic acid (RNA) sequencing analysis, has unveiled a landscape of widespread microRNA (miRNA) dysregulation in patients with ITP.^24^ miRNAs are small non‐coding RNA molecules, typically 20–22 nucleotides in length, that regulate gene expression by binding to target mRNAs and serve as important post‐transcriptional gene regulators. Studies show that miRNAs can regulate immune gene expression in a network‐like manner and shape immune‐cell phenotypes and functions, such as by modulating cell differentiation, activation and polarization.^25^ This makes miRNAs biologically plausible regulators of the major ITP immune axes, including T‐cell imbalances,^26^, ^27^ B‐cell‐related autoantibody production,^28^ macrophage phagocytic activity^29^ and thrombopoiesis defects.^30^, ^31^ Accordingly, the following sections will review ITP‐associated miRNAs across four areas: T cells, B cells, macrophages and platelet production (megakaryocytes/bone marrow) using a ‘discovery–validation–function’ evidence chain framework.

## MicroRNA‐MEDIATED EPIGENETIC REPROGRAMMING OF IMMUNE RESPONSES

### MicroRNA in T cells: Disrupted tolerance and plasticity

**FIGURE 2:**
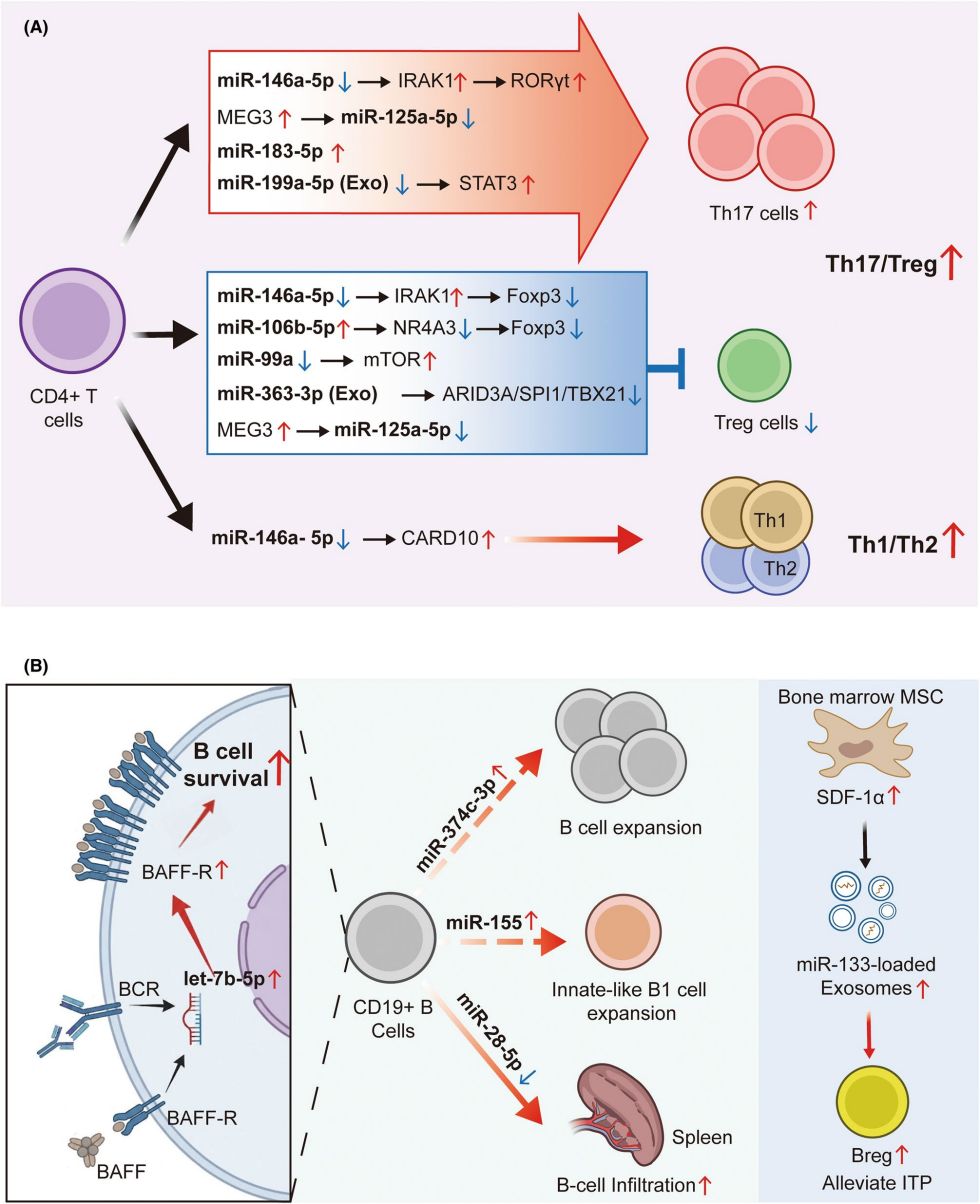
MicroRNA‐mediated dysregulation of T‐cell plasticity and B‐cell responses in ITP. (A) Dysregulated miRNAs in CD4^+^ T cells modulate key transcriptional and metabolic checkpoints (e.g. IRAK1, NR4A3, mTOR). These changes drive a pro‐inflammatory phenotype characterized by Th1/Th17 expansion and Treg deficiency. (B) In B cells, specific miRNAs relate to pathogenic behaviours, including enhanced survival, expansion of innate‐like B1 cells and splenic infiltration. Solid arrows represent pathways experimentally validated in ITP in vitro or in vivo, while dashed arrows indicate hypothetical links based primarily on expression profiling data that require further functional verification in ITP models. Additionally, MSC‐derived miR‐133‐loaded exosomes can support the maintenance of regulatory B (Breg) cells. BAFF, B‐cell activating factor; BAFF‐R, BAFF receptor; BCR, B‐cell receptor; Breg, regulatory B cell; Foxp3, forkhead box P3; ITP, immune thrombocytopenia; MSC, mesenchymal stem cell; RORγt, retinoic acid receptor‐related orphan receptor gamma t; Th, T helper cell; Treg, regulatory T cell.

T‐cell homeostasis, particularly the balance between pro‐inflammatory Th1/Th17 subsets and immunosuppressive Tregs, is essential for peripheral tolerance.^32^ In ITP, this balance is profoundly disturbed, characterized by an expanded Th1/Th17 compartment and numerically or functionally defective Tregs.^33^ High‐throughput profiling studies have identified distinct miRNA signatures in T cells in ITP, revealing at least 22 differentially expressed transcripts in peripheral blood T cells and over 500 in regulatory T cells.^34^, ^35^ Emerging evidence suggests that these dysregulated miRNAs are not just biomarkers, but they may actively regulate T‐cell plasticity and polarization through specific signalling pathways (Figure 2A).

The reciprocity between Th17 and Treg differentiation is governed by a complex network of lineage‐defining transcription factors, primarily *RORγt* and *Foxp3*.^36^ Several miRNAs have been identified as critical switches in this regulating process. Specifically, miR‐146a‐5p is a well‐known negative regulator of inflammation, and it is consistently decreased in the peripheral T cells of patients with ITP. By using CD4^+^ T cells from the peripheral blood mononuclear cells (PBMCs) of patients with ITP, an in vitro study indicated that miR‐146a‐5p deficiency leads to the disinhibition of its target *IRAK1*, a key node in the nuclear factor kappa‐light‐chain‐enhancer of activated B cells (NF‐κB) pathway.^37^ Another in vitro study showed that the consequent aberrant activation of inflammatory signals suppresses *Foxp3* expression while upregulating *RORγt*, thereby skewing the T‐cell phenotype towards Th17 dominance.^38^ Similarly, miR‐125a‐5p is downregulated in CD4^+^ T cells from patients with ITP compared to healthy controls, with its levels in ITP patients' serum positively correlating with haemoglobin concentration and platelet counts.^39^, ^40^ In vitro study, the downregulation of miR‐125a‐5p in ITP patients' CD4^+^ T cells is suggested to be mediated by upregulated long non‐coding RNA (lncRNA) MEG3.^39^ The MEG3/miR‐125a‐5p axis functionally mirrors the miR‐146a‐5p pathway, where loss of miR‐125a‐5p further destabilizes the Treg/Th17 ratio, favouring autoimmunity. Additionally, downregulation of miR‐99a was also observed in ITP patients' CD4^+^ T cells, and in vitro studies suggests that it is linked to the overactivation of the mechanistic target of rapamycin (mTOR) signalling pathway. Specifically, high mTOR activity promotes Th17 differentiation and reduces the stability of Tregs.^41^

While the loss of suppressive miRNAs impairs immune tolerance, the pathological upregulation of other miRNAs actively drives autoimmune responses. miR‐106b‐5p is significantly elevated in the PBMCs and CD4^+^ T cells from patients with ITP.^42^, ^43^ Functionally, it directly targets *NR4A3*, a nuclear receptor essential for *Foxp3* transcriptional stability. The repression of *NR4A3* by miR‐106b‐5p, coupled with reduced transforming growth factor beta (TGF‐β) signalling, impedes Treg differentiation and promotes Th17 expansion in vitro. Additionally, in vivo studies using a murine ITP model confirmed that silencing miR‐106b‐5p promotes Treg‐cell differentiation and elevates platelet counts.^42^ Furthermore, metabolic reprogramming, a rapidly evolving field in immunometabolism, appears to be under miRNA control. The upregulation of miR‐183‐5p was observed in ITP patients' CD4^+^ T cells and has been linked to the high level of Th17 activity in an in vitro study.^41^ Additionally, miR‐641 overexpression was linked to this imbalance in peripheral T cells from patients with ITP, and its in vitro knockdown successfully restored the Th17/Treg balance by targeting *STIM1* and *SATB1*.^44^

Recent advances have expanded the understanding of ITP pathogenesis from cell‐intrinsic defects to intercellular communication failures mediated by exosomes.^45^, ^46^, ^47^ miR‐199a‐5p is downregulated in plasma, splenocytes and PBMCs from patients with ITP. It functions as a potent repressor of *STAT3*, the master transcription factor for Th17 differentiation.^45^ Interestingly, miR‐199a‐5p‐loaded exosomes inhibited Th17 polarization in vitro and alleviated murine ITP in vivo, suggesting its systemic loss exacerbates the pro‐inflammatory environment. Plasma‐derived exosomes in ITP were enriched with miR‐363‐3p. In vitro assays demonstrated that these exosomes can be internalized by Tregs, where miR‐363‐3p targeted the ARID3A/SPI1/TBX21 axis.^48^ Disruption of this axis impaired the suppressive function of Tregs. These data suggest that miRNAs can spread immune dysfunction systemically, beyond their usual roles inside cells.

In addition to the Th17/Treg balance, the classical Th1/Th2 imbalance also plays a role in ITP pathogenesis.^49^ As previously discussed, miR‐146a‐5p exhibits multiple roles. Besides its effects on Tregs, its low levels also impact *CARD10* expression, which has been reported to suppress Th2 polarization and promote Th1 responses in vitro. Consequently, this creates a cytotoxic environment responsible for platelet destruction.^50^

Taken together, these findings support a multihit model where an abnormal miRNA expression profile plays a key role in ITP by influencing gene regulation and cell metabolism in T cells. Furthermore, miRNAs not only function intrinsically within T cells but can also be transmitted among cells via exosomes and mediate cell‐to‐cell communication. However, as highlighted in this section, although these studies aim to investigate the regulatory functions of miRNAs in T cells, the sample selection during the initial profiling phase varies significantly across cohorts. Moreover, studies incorporating robust in vivo validation remain in the minority. Therefore, standardizing methodological approaches is a crucial future direction for accurately characterizing miRNA expression and function within specific cell populations in ITP.

### MicroRNA in B cells: Hyperactivation, survival and regulatory failure

While T‐cell dysregulation provides the inductive signals, autoreactive B cells play an important role in ITP pathology through the secretion of platelet autoantibodies and antigen presentation.^51^ High‐throughput sequencing has revealed altered miRNA patterns in B cells that are correlated with disease activity in ITP. For instance, miR‐374c‐3p expression in peripheral CD19^+^ B cells was significantly altered in untreated patients with ITP compared with healthy controls and correlates with B‐cell frequency, suggesting a potential role in the expansion of the B‐cell pool.^52^ In addition to expression profiling, mechanistic studies suggested that miRNAs can drive B‐cell pathogenicity in ITP primarily by promoting cell survival and regulating B‐cell behaviour in the spleen (Figure 2B).

At the molecular level, miRNAs promote this autoreactivity primarily by enhancing cell survival. In ITP, let‐7b‐5p increased the resistance of B cells to apoptosis. Specifically, it upregulated the B‐cell activating factor (BAFF) receptor (BAFF‐R) and created a positive feedback loop that makes B cells more responsive to survival signals in vitro.^53^ Beyond survival pathways, miRNAs also drive the expansion of specific pathogenic subsets. Clinical profiling indicated that high levels of miR‐155 in CD19^+^ B cells of ITP patients are correlated with lower platelet counts and are specifically associated with the expansion of innate‐like B1 cells. Together, these miRNA signatures lowered the activation threshold of B cells and this may be associated with a loss of immune tolerance.^54^ In addition, the pathogenic impact of B cells depends on their spatial localization, a process also under miRNA control. Profiling of peripheral whole blood revealed decreased miR‐28‐5p in ITP patients. Mechanistically, in vitro assays confirmed that miR‐28‐5p directly targets Rap1b, and in vivo experiments demonstrated that restoring miR‐28‐5p effectively reduces pathogenic B‐cell infiltration and alleviates splenic inflammation in ITP mouse models.^55^ Finally, similar to T cells, intercellular communication by exosomes may contribute to tolerance restoration. In vitro assays demonstrated that upregulating stromal cell‐derived factor 1α (SDF‐1α) in mesenchymal stem cells (MSCs) enhances the enrichment of miR‐133 in secreted exosomes, thereby restoring impaired Breg populations from ITP patients.^56^ This implies that B‐cell defects in ITP are partially driven by impaired support from the bone marrow microenvironment. However, compared to the extensive research on T cells, in vivo functional studies investigating the role of miRNAs in ITP B cells remain scarce. Consequently, further in vivo models are urgently required to fully elucidate how miRNAs regulate B‐cell responses and the subsequent production of anti‐platelet autoantibodies within a complex immune microenvironment.

### MicroRNA in the monocyte–macrophage system: Phagocytic machinery and polarization dynamics

**FIGURE 3:**
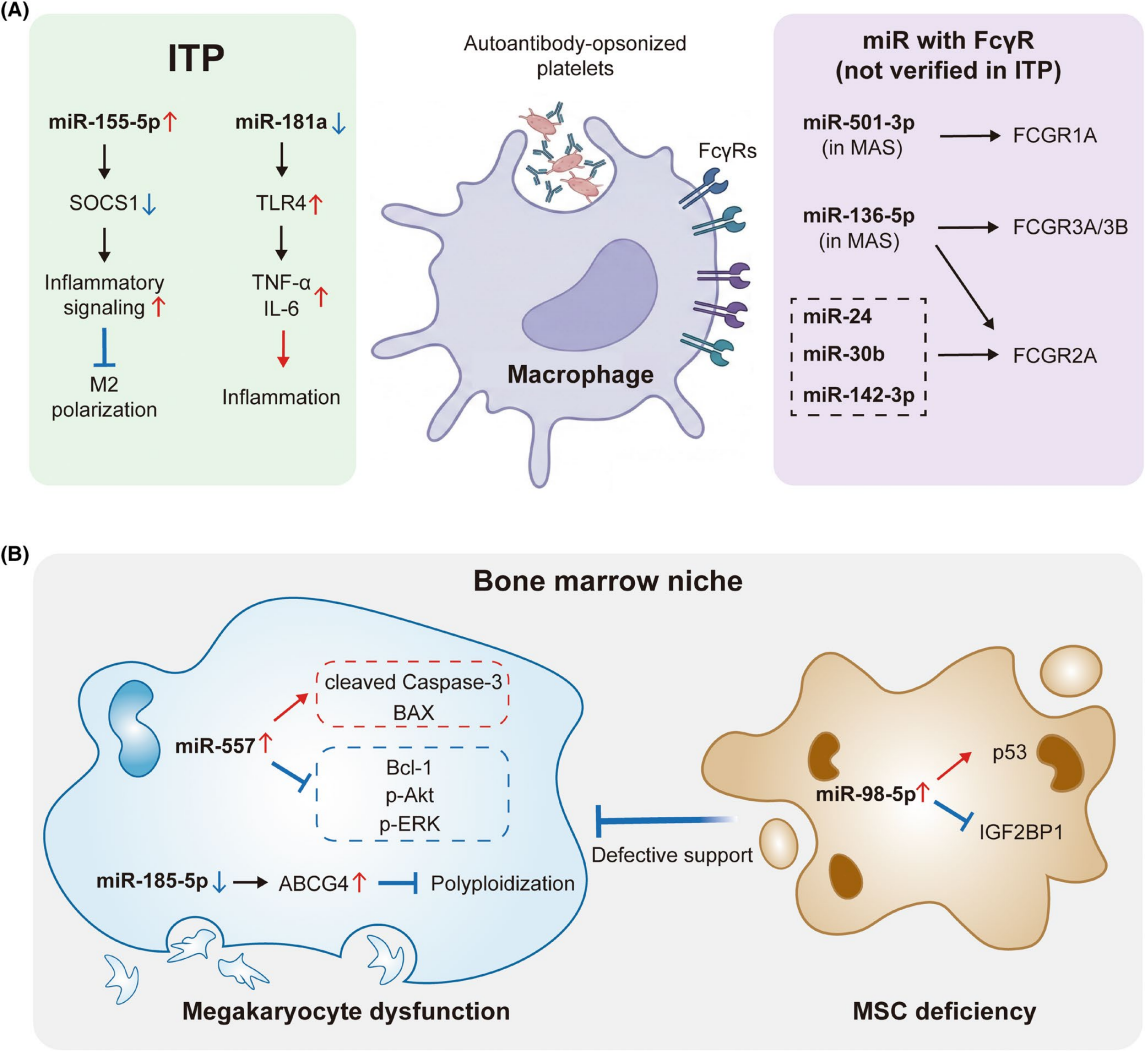
MicroRNA‐mediated modulation of macrophages and the bone marrow niche in ITP. (A) Macrophages exhibit a pro‐inflammatory M1 phenotype driven by abnormal levels of miR‐155‐5p and miR‐181a. The right panel illustrates candidate miRNAs potentially regulating FcγR expression but whose mechanistic roles have not been verified in ITP. (B) In the bone marrow, aberrant levels of miR‐185‐5p and miR‐557 in megakaryocytes arrest polyploidization and lead to dysfunction. Concurrently, upregulated miR‐98‐5p drives mesenchymal stem cell (MSC) deficiency, which impairs the supportive microenvironment for platelet production. FcγR, Fc gamma receptor; IL‐6, interleukin‐6; ITP, immune thrombocytopenia; MSC, mesenchymal stem cell; MAS, macrophage activation syndrome; TLR4, Toll‐like receptor 4; TNF‐α, tumour necrosis factor alpha.

Macrophages are the primary cells responsible for platelet destruction in ITP through ADCP. However, their role is not only limited to platelet clearance but also to help organize the inflammatory environment. In ITP, macrophages often skew towards the pro‐inflammatory M1 phenotype, and this polarization process is tightly regulated by specific miRNAs (Figure 3A). Profiling of patient‐derived PBMCs and macrophages revealed a significant upregulation of miR‐155‐5p. In vitro assays demonstrated that miR‐155‐5p directly targets *SOCS1*, and its inhibition promotes M2 polarization via the PD‐1/PD‐L1 pathway. Furthermore, in vivo experiments confirmed that silencing miR‐155‐5p shifts macrophages towards an M2 phenotype and effectively prevented thrombocytopenia in ITP mouse models.^29^ Acting as a protective counterbalance to these inflammatory drivers, miR‐181a worked to reduce inflammation, and it is found to be downregulated in PBMCs of ITP patients. In vitro assays demonstrated that miR‐181a targets *TLR4* to suppress inflammatory signalling. Moreover, in vivo experiments using ITP mouse models showed that restoring miR‐181a levels reduced *TLR4* expression and the secretion of tumour necrosis factor alpha (TNF‐α) and interleukin‐6 (IL‐6), thereby alleviating the hyper‐inflammatory state in ITP mouse models.^57^

While FcγR‐mediated phagocytosis is the primary pathophysiological process of platelet clearance, direct evidence linking specific miRNAs to FcγR modulation within the ITP context remains limited. However, data from biologically parallel conditions provide a compelling roadmap for future investigation. For example, in macrophage activation syndrome (MAS), miR‐136‐5p targeted *FCGR2A* and *FCGR3A/3B* (orthologues of mouse Fcgr3/4), while miR‐501‐3p targeted *FCGR1A* in activated macrophages.^58^ These findings demonstrated that miRNAs regulate the expression of FcγRs on monocyte/macrophage surfaces, a regulatory mechanism that is likely conserved in the pathogenesis of ITP. Furthermore, broader immunological studies identified a cluster of FcγR‐modulating miRNAs, including miR‐24, miR‐30b and miR‐142‐3p. In primary human macrophages and dendritic cells, transfection with these miRNA mimics resulted in a significant reduction of *FCGR2A*, *FCER1G* and *FCER2*. Notably, miR‐30b has been confirmed to post‐transcriptionally regulate *FCER1G*.^59^ In addition, in vitro evidence showed that miR‐181a negatively regulated FcγR‐mediated sFlt‐1 production, a process antagonized by NF‐κB signalling, which suggests a potential role for this axis in modulating systemic inflammation.^60^ Although these interactions await specific validation in ITP macrophage models, they represent high‐priority candidates. Given that FcγR‐mediated phagocytosis is a pivotal pathogenic mechanism in ITP, future research should prioritize comprehensive in vitro and in vivo functional experiments. Such studies are essential to validate the exact roles of these dysregulated miRNAs in driving macrophage dysfunction and subsequent thrombocytopenia.

### MicroRNA in the bone marrow niche: Impaired megakaryopoiesis and defective support

The pathogenesis of ITP is not limited to platelet destruction but is also driven by impaired platelet production within the bone marrow niche.^61^, ^62^ Megakaryopoiesis is the complex process of forming platelets, involving endomitosis and cytoplasmic maturation. This process is critically dependent on stable miRNA levels. Providing the most direct functional evidence in ITP, validated through both clinical profiling and in vivo models, elevated miR‐557 promoted megakaryocytes (MKs) apoptosis by suppressing protein kinase B/extracellular signal‐regulated kinase (Akt/ERK) signalling and changing the Bcl‐2/BAX balance. It's in vivo inhibition effectively restores megakaryopoiesis and platelet counts in ITP rats (Figure 3B).^63^ Beyond this direct evidence, other miRNAs are implicated through in vitro and secondary models. In in vitro models mimicking the ITP microenvironment, miRNA patterns in MKs were significantly altered and were bioinformatically predicted to affect pathways controlling cell development metabolism.^63^ Furthermore, in virus‐induced in vitro models of secondary thrombocytopenia, miR‐185‐5p downregulation leads to the overexpression of *ABCG4*, which arrests MK polyploidization—a prerequisite for thrombopoiesis.^64,^, ^65^ Finally, several other targets represent important areas for future research. Molecules such as miR‐150, miR‐146a, miR‐15a and miR‐125b‐2 are known to be essential for normal megakaryopoiesis. However, few studies have tested them directly in ITP, which represents an important area for future research.^66^

Megakaryocytes do not exist in isolation; their maturation relies on paracrine support from MSCs. In ITP, MSCs exhibit distinct morphological and functional defects, often termed ‘MSC deficiency’, which compromises their ability to nurture MKs.^67^ Clinical profiling identified highly upregulated miR‐98‐5p in primary ITP‐MSCs. In vitro, this upregulation drove MSC deficiency by targeting *IGF2BP1* and impeding p53 degradation experiments. This double hit induces MSC apoptosis, compromising the marrow microenvironment. In vivo validations confirmed this effect, miR‐98‐5p‐driven apoptosis actively impaired the therapeutic and immunomodulatory functions of MSCs in ITP mice.^68^ Beyond the bone marrow niche, the intrinsic instability of platelets also contributes to thrombocytopenia. Despite being anucleate, platelets harbour a rich miRNA repertoire. Recent clinical profiling has identified specific miRNA patterns in ITP patient's platelets compared to healthy controls, including hsa‐miR‐548a‐5p, hsa‐miR‐30a‐3p and hsa‐miR‐765. Bioinformatic analyses predicted that these miRNAs target pathways involved in platelet apoptosis and adhesion defects.^69^ However, there is a lack of functional validation to confirm the specific roles of these miRNAs in platelet apoptosis. Investigating these platelet‐resident miRNAs offers a promising way for developing non‐invasive biomarkers that reflect the stress in the bone marrow during platelet production.

## TOWARDS CLINICAL TRANSLATION: THE POTENTIAL OF CIRCULATING MicroRNAs AS NON‐INVASIVE BIOMARKERS

Currently, ITP remains a diagnosis of exclusion, lacking specific gold standard biomarkers.^70^ The high stability of circulating miRNAs in serum, plasma and exosomes makes them ideal candidates for a ‘liquid biopsy’. Clinical research has focused on establishing miRNA‐based diagnostic models based on these miRNA patterns^47^ (Table 1). Several single miRNAs have demonstrated high diagnostic sensitivity. For instance, miR‐106b‐5p shows strong performance in primary ITP from healthy controls with an area under the curve (AUC) of 0.92,^42^ while upregulated serum let‐7a‐5p exhibits near‐perfect accuracy in paediatric ITP (AUC 0.995; sensitivity 100%, specificity 96.8%).^70^ Additionally, plasma miR‐199a‐5p (61 ITP vs. 28 HC) has been identified as a promising biomarker for primary ITP with an AUC of 0.718.^71^ Beyond initial diagnosis, certain miRNAs are effective in staging; for instance, miR‐200c‐3p (AUC 0.718) and miR‐92a‐3p (AUC 0.83) are particularly useful for identifying chronic ITP phases.^42^

**TABLE 1:** Summary of microRNAs in ITP categorized by evidence strength, cellular source and clinical biomarker potential.

miRNA	Sample/cell type	Evidence strength	Clinical biomarker
miR‐146a‐5p	PBMCs	Profiling only^37^, ^38^	
	CD4^+^ T cells	In vitro function^37^, ^38^, ^50^	
miR‐125a‐5p	Serum	Profiling only^40^	Prognostic prediction (responses to corticosteroids)
	CD4^+^ T cells	Profiling + in vitro function^39^	
miR‐99a	CD4^+^ T cells	Profiling + in vitro function^41^	
miR‐106b‐5p	PBMCs	Profiling only^43^	Diagnostic biomarker (primary ITP, chronic ITP)
	CD4^+^ T cells	Profiling + in vitro function + in vivo function^42^	
miR‐183‐5p	CD4^+^ T cells	Profiling + in vitro function^41^	
miR‐641	CD4^+^ T cells	Profiling + in vitro function^44^	
miR‐199a‐5p	Spleen cells	Profiling + in vitro function + in vivo function^45^	
	Plasma		Diagnostic biomarker (primary ITP) Prognostic prediction (responses to TPO‐RAs)
miR‐363‐3p	Plasma exosomes	Profiling + in vitro function^48^	
miR‐374c‐3p	CD19^+^ B cells	Profiling only^52^	
let‐7b‐5p	CD19^+^ B cells	Profiling + in vitro function^53^	
miR‐155	CD19^+^ B cells	Profiling only^54^	Prognostic prediction (refractory or higher relapse risk)
miR‐28‐5p	Peripheral blood	Profiling only^55^	
	CD19^+^ B cells	In vitro function + in vivo function^55^	
miR‐133	CD19^+^ B cells	In vitro function^56^	
miR‐155‐5p	PBMCs, Macrophages	Profiling + in vitro function + in vivo function^29^	
miR‐181a	PBMCs	Profiling only^57^	
	Macrophages	In vitro function + in vivo function^57^	
miR‐557	Plasma	Profiling only^63^	
	MKs	In vitro function + in vivo function^63^	
miR‐98‐5p	MSCs	Profiling + in vitro function + in vivo function^68^	
miR‐200c‐3p	PBMCs		Diagnostic biomarker (chronic ITP, chronic ITP vs. primary ITP)^43^
miR‐92a‐3p	PBMCs		Diagnostic biomarker (chronic ITP, chronic ITP vs. primary ITP)^43^
let‐7a‐5p	Serum		Diagnostic biomarker (paediatric ITP)^71^
miR‐130a	PBMCs		Prognostic monitoring (recovery of platelet counts)^74^
4‐miRNA signature (miR‐144‐3p, miR‐1275, miR‐3141, miR‐3162‐3p)	Plasma		Diagnostic biomarker (primary ITP)^73^
3‐miRNA signature (miR‐199a‐5p, miR‐33a‐5p, miR‐374b‐5p)	Plasma		Diagnostic biomarker (primary ITP)^72^

Abbreviations: ITP, immune thrombocytopenia; MKs, megakaryocytes; MSCs, mesenchymal stem cells; PBMCs, peripheral blood mononuclear cells; TPO‐RAs, thrombopoietin receptor agonists.

To further enhance diagnostic specificity and the AUC, researchers have developed multi‐miRNA signatures using plasma samples. A notable example is a 4‐miRNA panel (miR‐144‐3p, miR‐1275, miR‐3141 and miR‐3162‐3p) which achieved an AUC of 0.836 for primary ITP (37 ITP vs. 32 HC). While a traditional 5‐platelet autoantibody (AAbs) panel yielded an AUC of 0.875, integrating this 4‐miRNA signature with the 5‐AAbs panel significantly improved the diagnostic accuracy to an AUC of 0.948.^72^ Similarly, another 3‐miRNA signature (miR‐199a‐5p, miR‐33a‐5p and miR‐374b‐5p) also demonstrated high efficacy with an AUC of 0.865 (61 ITP vs. 28 HC).^73^ These findings suggest that, while circulating miRNAs currently serve as powerful adjunct tools rather than stand‐alone replacements, their superior sensitivity and the ability to distinguish ITP subtypes significantly complement existing exclusion‐based criteria. By combining miRNA signatures with traditional antibody assays, clinicians can achieve higher diagnostic confidence and better differentiate ITP from other thrombocytopenic conditions.

Besides diagnosis, miRNAs serve as valuable tools for personalized ITP management through therapy prediction and longitudinal monitoring. For pretreatment prediction, high baseline serum miR‐125a‐5p (45 newly diagnosed ITP vs. 45 HC) predicted favourable sensitivity to corticosteroids.^39^ Similarly, in CD19^+^ B cells, low levels of miR‐155 (30 newly diagnosed ITP vs. 25 in remission vs. 25 HC) correlate with favourable outcomes, while sustained elevation indicates a refractory course or higher relapse risk.^54^ For second‐line treatments, regression analyses have shown that plasma miR‐199a‐5p and miR‐221‐3p (42 ITP vs. 41 HC) are independent predictors of platelet responses to TPO‐RAs.^46^ Regarding therapeutic monitoring, specific miRNAs reflect disease remission or progression. In active chronic ITP (16 active chronic ITP vs. 14 HC), effective treatment leads to the restoration of PBMCs' miR‐130a and *TGF‐β1* levels, mirroring the recovery of platelet counts.^74^ Thus, integrating these markers into clinical practice could optimize drug selection and reduce the financial burden by identifying non‐responders early.

## CHALLENGES AND FUTURE PERSPECTIVES: FROM MOLECULAR LANDSCAPES TO CLINICAL IMPLEMENTATION

It appears that miRNAs may be essential upstream regulators of the immune imbalance in ITP. In peripheral blood, miRNAs regulate the functions of T cells, B cells and macrophages and are linked to cytokine release and the destruction of platelets. In the bone marrow, abnormal miRNA levels also interfere with how megakaryocytes form platelets. Their potential as non‐invasive biomarkers may help transform the current diagnosis of exclusion into a molecularly defined precision diagnostic approach. However, the clinical translation of these findings is currently hindered by the fragmentation of data. Most existing studies are restricted to single‐centre, small‐cohort designs and lack external validation. Furthermore, the absence of a dedicated, unified database for ITP‐associated non‐coding RNAs precludes the standardization of results across studies. This fact makes it difficult to normalize results thus impeding the execution of large‐scale meta‐analyses required to establish robust and evidence‐based consensus miRNA signatures.

There is also a major problem in the discordance of sample selection and processing. Current studies exhibit significant heterogeneity, with studies utilizing serum, plasma or isolated PBMCs without standardized operating procedures. For example, during serum preparation, the coagulation process triggers the release of a massive repertoire of miRNAs from platelets and extracellular vesicles,^75^, ^76^ which can mask the subtle immune‐related signals originating from, for example, lymphocytes. Consequently, plasma (particularly platelet‐poor plasma) typically reflects the circulating repertoire more accurately than serum. The lack of control over these pre‐analytical steps reduces data comparability and reproducibility. Therefore, a global consensus guideline for handling samples in ITP research needs to be established. Furthermore, identified miRNAs often vary significantly across similar ITP cohorts due to methodological heterogeneity, small sample sizes and candidate selection bias. First, analysing diverse biological compartments, such as PBMCs, isolated CD4^+^ T cells, CD19^+^ B cells or macrophages, naturally yields distinct profiles. Second, reliance on small patient cohorts severely reduces statistical power, amplifying non‐reproducible, cohort‐specific variations. Finally, significant bias occurs during functional validation. While high‐throughput profiling often reveals some overlapping dysregulations across studies, researchers typically select only one or two top candidates for downstream mechanistic assays. Consequently, literature focusing on functionally verified miRNAs creates the illusion of completely discordant profiles, whereas these functionally verified candidates may be chosen from a broader, overlapping pool.

In addition, many studies only describe different miRNA expression profiles between patients and healthy controls without functional validation.^77^, ^78^ While such associative studies are foundational, they fail to establish causality. The field urgently needs to transition towards functional and mechanistic exploration. Moreover, translating miRNA therapeutics into clinical practice faces significant delivery challenges. These treatments mainly use synthetic mimics to restore miRNAs or single‐stranded inhibitors of miRNA (antimiRs) to block them.^79^ However, unprotected RNAs are quickly degraded by cellular and serum RNases. To improve stability, researchers use chemical backbone modifications, such as 2′‐O‐methylation and locked nucleic acids (LNAs), or use advanced delivery vectors, including lipid nanoparticles (LNPs) and engineered exosomes, to deliver miRNA mimics or antimiRs.^80^, ^81^, ^82^ For ITP, an ideal delivery system must cross the blood‐bone marrow barrier to target the marrow microenvironment and selectively target specific immune cells. This precision is vital to prevent off‐target toxicities. Therefore, developing safe and targeted delivery systems is a crucial step for the clinical translation of miRNA‐based ITP therapies, and it remains a key direction for future research.

## AUTHOR CONTRIBUTIONS

ZL involved in literature search and writing first draft; JR, LG, WG, PX, HW and RK edited the draft; DP edited the draft and figures; JWS involved in student supervision, editing draft and financial support.

## CONFLICT OF INTEREST STATEMENT

The authors have no conflicts of interest to disclose.

## Data Availability

Data sharing not applicable to this article as no datasets were generated or analysed during the current study.
